# IGF-Binding Protein 2 – Oncogene or Tumor Suppressor?

**DOI:** 10.3389/fendo.2015.00025

**Published:** 2015-02-27

**Authors:** Adam Pickard, Dennis J. McCance

**Affiliations:** ^1^Centre for Cancer Research and Cell Biology, Queen’s University, Belfast, UK

**Keywords:** IGF1, IGFI, IGF2, IGF-II, IGFBPs, IGFBP2, oncogenes, tumour suppressor

## Abstract

The role of insulin-like growth factor binding protein 2 (IGFBP2) in cancer is unclear. In general, IGFBP2 is considered to be oncogenic and its expression is often observed to be elevated in cancer. However, there are a number of conflicting reports *in vitro* and *in vivo* where IGFBP2 acts in a tumor suppressor manner. In this mini-review, we discuss the factors influencing the variation in IGFBP2 expression in cancer and our interpretation of these findings.

## Introduction

Insulin-like growth factor binding protein 2 is a member of the family of six IGF-binding proteins, IGFBP1–6, these proteins bind with high-affinity to IGF1 and IGF2 (1); in addition, there is also a low affinity for insulin (2). Other IGF-binding proteins such as the CCN family retain the name as IGFBPs, although these have ~100-fold lower affinity for IGF1 and IGF2 (1, 3, 4). IGFBP2 has the ability to bind insulin, IGF1 and IGF2 with an increased affinity for the latter (2, 5). IGFBP2 and the other family members have been proposed to suppress tumor development through binding IGFs preventing binding to their receptor and thereby preventing IGF driven tumorigenesis (6). However, despite this suppressive function, there are also studies that demonstrate oncogenic functions including promoting proliferation, driving invasion, and suppressing apoptosis (7). These effects appear to be independent of its ability to bind the IGFs and instead promote invasion and proliferation through interaction with integrins (8, 9).

Insulin-like growth factor binding protein 2 has been proposed as a potential biomarker in various cancer types, including gliomas (10–18), prostate (19–27), ovarian (28–33) colorectal (34–40) and acute myeloid leukemia (13, 41, 42), acute lymphoblastic leukemia (43, 44) pancreatic (45–48), lung (49–52), cervical cancer (53), breast (54, 55) including triple negative breast cancer (56), liver (57–60), head and neck (61), rhabdomyosarcoma (62), non-seminomatous germ cell cancer (63), gastric (64), synovial sarcoma (65), adrenocortical (66), and Wilm’s tumors (67). These studies have identified changes in tissue levels or circulating plasma levels of IGFBP2 protein, although it is not yet clear whether tumor or circulating levels are better as cancer biomarkers as each approach provides conflicting data. Despite this extensive investigation, IGFBP2 has not yet been implemented as a cancer biomarker due to contradictory findings as to whether IGFBP2 is up- or down-regulated in different cancers. There are multiple factors, which can influence IGFBP2 expression and which can alter our interpretation of these findings; in the following sections, we propose that future experiments should aim to account for these factors in order to improve our understanding of IGFBP2’s role in cancer.

## Metabolic/Dietary Modulation of IGFBP2

Insulin-like growth factor binding protein 2 is being evaluated as a potential cancer biomarker as it is a secreted protein that is readily detected in a patients’ plasma; higher levels have repeatedly been associated with disease severity in prostate cancer and gliomas. However, there are also inconsistent finding, which makes evaluation of IGFBP2’s oncogenic/tumor suppressive role difficult and also questions the suitability of IGFBP2 as a biomarker (68). Therefore, it is important to consider whether there are confounding factors, which obscure our interpretation. Plasma levels of IGFBP2 are known to inversely correlate with obesity/body mass index (BMI) (69–76), and insulin resistance. In addition, diet (77), fasting (78), age, and physical activity (79) also influence IGFBP2 plasma levels. If not included within the study design, these factors are likely to hinder our evaluation of IGFBP2 as a biomarker. The importance of combining these parameters along with measurements of IGFBP2 levels is demonstrated by Probst-Hensch et al. (80). By combining BMI and the levels of IGFBP2 and IGFBP3 in assessment of breast cancer patient survival, the authors demonstrated that without correction for BMI neither IGFBP2 nor IGFBP3 were predictive of overall survival, whereas inclusion of BMI identified that higher IGFBP2 levels to be a beneficial prognosticator. This is an interesting finding as it is generally considered that IGFBP2 is oncogenic in breast cancer (55, 81); however, these other studies did not correct for factors such as BMI. Similarly, the influence of obesity has been examined with respect to the levels of IGFBP2, with lower levels observed in obese patients, which has been proposed to be a result of hyperinsulinemia, with insulin suppressing IGFBP2 expression (82). This has been linked with the subsequent increase in proliferation mediated by free-IGFs and thereby increasing the risk of cancer in obese individuals (82). In some respects, this interpretation is complemented by data in IGFBP2 transgenic mice, which have reduced body mass (83) and incidence of colorectal adenomas in experimental models (84).

## Regulation of IGFBP2 Expression at a Cellular Level

Given the difficulties in accurately associating plasma levels of IGFBP2 with cancer patient prognosis or disease progression, as well as reports demonstrating a lack of concordance between tumor immunohistochemistry (IHC) and serum levels of IGFBP2 (12, 52, 85–87), it may be more meaningful to assess the expression of IGFBP2 within the tumor itself. IGFBP2 is mainly expressed by the liver, adipose, and reproductive tissues and tissues of the central nervous system (4). However, IGFBP2 is also expressed in a wide range of other tissue types, including both epithelial and mesenchymal cells [see Ref. (88), proteinatlas.org (89), and encode (90)], therefore the local levels of IGFBP2 are likely to play an important part in how the tissue responds to circulating IGFs. Approaches to detect changes in protein or RNA levels of IGFBP2 within tumors have been used to hint at whether IGFBP2 might act in a tumor suppressive or oncogenic manner, however, within a given tumor type the expression of IGFBP2 can vary greatly, so below we examine the mechanisms, which regulate IGFBP2 expression in tumors.

### Promoter methylation

Promoters, including the IGFBP2, can become hyper- or hypo-methylated relative to the normal state during tumorigenesis. The IGFBP2 promoter has been shown to be hypermethylated, in a subset of tumors, including 8% of small cell lung carcinomas (86), >20% of renal cell carcinomas (91), ~30% squamous cell lung cancers (86), 40% of colorectal cancers (92), >70% lung adenocarcinomas (86), and 75% hepatomas (93). The hypermethylation results in reduced IGFBP2 expression, as demonstrated by Yazawa et al. with good correlation between IGFBP2 promoter methylation and IGFBP2 protein expression detected by IHC (86). The low expression of IGFBP2 observed in some glioblastoma cell lines, is associated with methylation of the IGFBP2 promoter and can be restored with 5-azacytadine treatment suggesting regulation by DNA methyltransferase 1 (DNMT1) (94). Also, DNMT3L knockdown in embryonic stem cells results in elevated IGFBP2 expression (95), suggesting promoter methylation is an important regulator of IGFBP2 expression. The observation that IGFBP2 is hypermethylated in lung and liver cancers is somewhat surprising as elevated plasma levels have been described for these diseases (57, 87); however, the higher plasma levels of IGFBP2 may represent a systemic response to cancer, or the confounding factors described above. Further assessment of the inactivation of the IGFBP2 locus will be greatly aided by The Cancer Genome Atlas project (http://cancergenome.nih.gov/).

### Normal versus aberrant regulation

Insulin-like growth factor binding protein 2 expression is greatly influenced by hormonal factors and both positive and negative regulators of IGFBP2 expression have been described, as reviewed in Ref. (88). Interestingly, the same hormone can have differing effects depending upon the tissue. As an example, estradiol (E2) acts on the hippocampus to increase IGFBP2 expression (96) whereas in the cortex IGFBP2 expression is reduced (97). Estradiol also reflects an interesting example of how we interpret the role of IGFBP2 in cancer, as previously described, IGFBP2 is generally considered to be oncogenic in breast cancer; however, IGFBP2 expression, *in vivo*, is induced by estradiol in normal breast tissue (98), whereas in the rat mammary adenocarcinoma, R3230AC, IGFBP2 expression is reduced following E2 treatment (99). In the breast cancer cell line, MCF7 IGFBP2 expression is also elevated by E2 (100), suggesting that this cell line is responding in the same way as normal tissue. Estrogen receptor (ER) status directly correlates with IGFBP2 expression in breast cancer, with ER-positive cancers having higher levels of IGFBP2 than ER-negative cancers (80, 101, 102), and therefore the high levels of IGFBP2 may simply reflect a functional ER pathway. In order to better understand whether regulation of IGFBP2 expression is de-regulated in cancer, it will be important to further establish how it is regulated in the physiological setting.

### Regulation by proteases

In addition to the aforementioned disconnect between plasma levels and tumor levels of IGFBP2, there are also reports demonstrating that IGFBP2 mRNA and protein levels do not always correlate (103). Protease cleavage of IGFBP2 could be partially responsible for the lack of association, and also help to explain the differential findings of IGFBP2’s role in cancer. There are a number of proteases, which cleave the mature IGFBP2 peptide, these include calpain (104), pappalysin A (105), kallikrein-2 (106), and MMP7 (107). Plasmin and MMP1 also cleave IGFBP2; however, the cleavage sites have not been defined (108). In addition, IGFBP2 is also cleaved at additional sites; however, the responsible proteases have not been identified (109). IGFBP2 cleavage reduces its affinity for IGFI and IGF2 (110–112), allowing IGFs to mediate their functions through the IGF1R. Thus, it is important to reflect on the nature of IGFBP2 in carcinogenesis as calpain (113), MMP1 (114), MMP7 (114), and kallikrein-related peptidases (115) have also been described to be elevated or activated in various cancer types and may lead to inactivation of the protein. Many reagents used to detect the IGFBP2 protein are not able to distinguish whether IGFBP2 is in its full-length form. Fragments of IGFBP2 can be detected by the antibodies often used for immunohistochemical detection of IGFBP2 (110). To our knowledge, only one report has assessed IGFBP2 expression with multiple antibodies, targeting the C and N termini of IGFBP2. This work demonstrated a correlation between a cleaved N-terminal fragment of IGFBP2 and the protease ADAMTS1 in glioblastoma (116) suggesting IGFBP2 is not in a full-length form and therefore may be unable to bind IGFs.

Insulin-like growth factor binding protein 2 cleavage may result in enhanced proliferation mediated by IGFs but it has also been proposed that the cleaved fragments of IGFBP2 have additional roles. C-terminal fragments of IGFBP2 are able to promote proliferation of rat chondrocytes (117), and a recent report, which demonstrates that nuclear IGFBP2 can drive VEGF expression demonstrated that a nuclear localization sequence (NLS) is present in the linker region of IGFBP2 (118); interestingly, this region is the site of many cleavage events and may lead to exposure of the NLS.

The importance of establishing whether IGFBP2 is cleaved in cancer tissue has recently been demonstrated by Soh et al. (108), where a protease-resistant IGFBP2 was more effective at inhibiting IGF1-induced proliferation of the MCF7 cell line than wild-type IGFBP2. Interestingly, protease resistant IGFBP2 was also able to suppress tumor growth *in vivo*. Furthermore, the MCF7 cell line is known to secrete low levels of full-length IGFBP2 compared to other cancer cell lines even though the mRNA and intracellular protein levels of IGFBP2 are comparable (119), suggesting high levels of proteolytic degradation.

Reflecting on the cleaved status of IGFBP2 in cancer may also help to explain some of the contradictory evidence regarding its tumor suppressive/oncogenic role in carcinogenesis. For example, the elevated expression of the IGFBP2 protein observed in various cancers may represent a response to suppress tumor growth, mediated by IGFs; however, pro-tumorigenic proteases may ultimately inactivate this response. Enhanced proteolysis of IGFBP2 has been described in proliferating cells, and can be inhibited by targeting proteases (120). Therefore, the observation that a protease-resistant IGFBP2 can suppress tumor growth (108) creates the exciting opportunity to target proteases and potentially restore the suppressive actions of IGFBP2.

### Intra-tumor variability

In addition to the variation of IGFBP2 expression between different tumors, there is also further variation of IGFBP2 expression levels, within an individual tumor. While high grade glioblastoma, which are frequently observed to have elevated IGFBP2 level, also have regions of reduced IGFBP2 expression, which have been shown to be at the invasive front of the tumor (121). Therefore, this data would suggest that IGFBP2 loss correlates with enhanced invasive potential, as observed in other models (59). Interestingly, this is also supported by an *in vivo* model of malignant brain tumor growth and invasion, where rapidly growing non-invasive tumors have high levels of IGFBP2 compared to invasive tumors, which had low/undetectable IGFBP2 levels (122). Furthermore, the oxygenation of a tumor varies depending on blood supply to different regions of the tumor, hypoxia has been shown to regulate IGFBP2 expression (88, 123–126) and should also be considered when scoring IGFBP2 expression in tumor samples.

### Regulation of IGFBP2 expression by tumor suppressors and oncogenes

There are a number of additional factors, which are known to influence IGFBP2 expression, and therefore how we evaluate its role in cancer. There are now a number of reports, which demonstrate a connection between PTEN levels and those of IGFBP2 (127–130). *PTEN* mutation in glioma is associated with high levels of IGFBP2 (130) suggesting that IGFBP2 could be a marker of *PTEN* mutation. Similarly, mutation of *KRAS* also induces IGFBP2 expression (131). IGFBP2 expression has also been shown to correlate with *TP53* mutational status. While it has been demonstrated that wild-type p53 is required for IGFBP2 induction following irradiation (132), the regulation of IGFBP2 by p53 mutations is, at present, unclear. In breast cancer, p53 mutation is associated with reduced secretion of IGFBP2 (133), whereas in glioblastoma, mutant p53 is associated with high levels of IGFBP2 (94). In addition, Ras mutation, loss of p53 and radiation-induced DNA damage drives a senescence-associated secretory phenotype (SASP), of which, elevated IGFBP2 secretion is a component (134). This is also supported by evidence that mRNA expression is also elevated in senescence (135–137). Together these findings suggest that the variation in IGFBP2 expression in tumors may reflect the status of tumor suppressors, oncogenes, and/or use of radiation therapy, therefore, the tumor suppressive/oncogenic potential of IGFBP2 should now be evaluated in the context of each mutation in order to demonstrate whether or not IGFBP2 has a functional role.

## Correlation of IGFBP2 Expression with Cancer Progression

It has been established that enhanced expression of IGFBP2 is associated with the progression of tumorigenesis in prostate (27), breast cancer (138), and glioma (7); however, when the expression of IGFBP2 is examined further it appears that there are certain cancer types or subpopulations of cancers, which develop with little or no expression of IGFBP2. This latter finding goes against the hypothesis of IGFBP2 being a driver of tumorigenesis, and suggests that loss of IGFBP2 may also be an important event in tumors. A detailed examination of ovarian cancers observed that IGFBP2 expression did not correlate with stage of disease (139), with similar proportions of tumors having low or high expression of IGFBP2 at all stages. Interestingly, in the same study, the authors demonstrate that the expression of IGFBP2 is related to the type of ovarian cancer, for example, 80% of serous carcinoma had elevated IGFBP2 whereas 80% of clear cell carcinoma had low IGFBP2 levels. Similarly, in lung cancer, IGFBP2 is found to be overexpressed in the majority of small cell lung carcinomas but reduced in the majority of lung adenocarcinomas (86). Thus, it is important to consider whether these results demonstrate a correlation of IGFBP2 driving tumorigenesis or are, in fact, reflective of tumor stage/subtype. These findings suggest that cancers can develop both in the presence and absence of IGFBP2 and as cancer sub-types become further defined it will be important to evaluate how IGFBP2 expression is regulated among these different groups. Evaluation of IGFBP2’s role in each group will then provide a better understanding of it oncogenic and tumor suppressive potential.

## Therapeutic Response and IGFBP2

Evaluation of IGFBP2 as a biomarker in the treatment of cancer has also generated a dichotomy in how IGFBP2 is perceived. In some cases, IGFBP2 is found to be a marker of response to therapy but then others have demonstrated that high levels confer resistance. In the case of IGF1R targeted therapies, McCaffery et al. (47) demonstrated that there was better response to IGF1R targeted therapy in patients with low IGFBP2 levels, than patients with high IGFBP2 and similar results were found in a mouse model where inhibition with the IGF1R inhibitor BMS-536924, had no effect in reducing growth of cells expressing high levels of IGFBP2 (140). However, down-regulation of IGFBP2 in rhabdomyosarcoma is associated with resistance to IGF1R therapy (141). In response to other therapeutic strategies, tumor samples from patients receiving chemotherapy/radiotherapy have been shown to have elevated IGFBP2 levels and suggest that IGFBP2 is a potential marker of response to these treatments (134) and may be due to a pro-apoptotic function of IGFBP2 (142). However, high levels of IGFBP2 are also linked with resistance to other therapeutics (143–146) and high levels of IGFBP2 following radio/chemotherapy also correlate with poor survival in elderly patients with glioblastoma (147). Therefore, further evaluation of IGFBP2 in response to therapy is an area, which requires further investigation.

## Differential Functions of IGFBP2

The binding to IGF1 and IGF2 is common to *in vitro* models, which demonstrate either oncogenic or tumor suppressive functions of IGFBP2 (148). In addition to repressing IGF signaling, IGF-independent functions have also been described. Overexpression or addition of recombinant IGFBP2 activates integrin complexes with integrins α5 and β1 mediating the effects of IGFBP2 (142, 149–151). Through these pathways IGFBP2 reduces adhesion and promotes proliferation and invasion, some of these activities are also observed in cell lines that lack the IGF1-receptor (142) and therefore helps to explain the dichotomy of IGFBP2 functions *in vitro* and may also explain the association of high levels of IGFBP2 with high grade tumors, despite its inhibitory effect on IGF signaling. It is important to note that these observations have been made following addition of 100–2000 ng/mL recombinant IGFBP2, while these levels are within the physiological range of human plasma these are greater than the levels secreted by cell lines in culture (151).

## Conclusion

While there is conflicting evidence as to whether IGFBP2 is tumor suppressive or oncogenic, this mini-review highlights a number of factors, which influence our interpretation of these findings. Through detailing factors, which influence IGFBP2 expression alongside measurements in patient samples our current understanding of its role in cancer could be improved. So far, it is clear that IGFBP2 has profound effects on cancer cell biology both *in vitro* and *in vivo* and therefore warrants further evaluation of IGFBP2 either as a biomarker or even as a therapeutic. The tumor suppressive functions of IGFBP2 align with its ability to bind IGFs while its oncogenic properties of IGFBP2 appear to be IGF-independent. In the future assessment of IGFBP2’s role in cancer, it will be important to consider (i) should IGFBP2 levels be assessed in plasma or the tumor/tumor microenviroment? and (ii) can we assess the activity status of IGFBP2 alongside expression levels? either through measuring IGFBP2 cleavage or through development of a functional assay. Further avenues will also likely assess whether IGFBP2 is a predictive biomarker for therapeutic response.

## Conflict of Interest Statement

The authors declare that the research was conducted in the absence of any commercial or financial relationships that could be construed as a potential conflict of interest.
